# Health disparities affecting LGBTQ+ populations

**DOI:** 10.1038/s43856-022-00128-1

**Published:** 2022-06-09

## Abstract

Dr. Jessica N. Fish is an Assistant Professor in the Department of Family Science, University of Maryland Prevention Research Center, University of Maryland, USA. Her research seeks to promote the positive development and health of LGBTQ+ people and their families. In this Q&A, Dr. Fish provides insight into the health disparities that affect LGBTQ+ populations, with a particular focus on mental health and the development of LGBTQ+ youth, and important research and policy considerations in this area.

**Figure:**
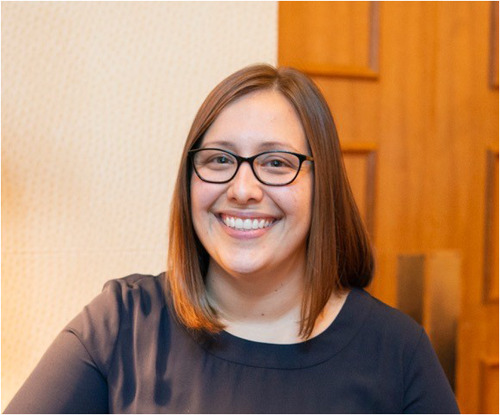
Jessica N. Fish

What kind of health issues disproportionately impact LGBTQ+ people at different points in their lives?

There is now a compelling body of science that documents elevated risk for a host of poor mental health and substance use outcomes among LGBTQ+ populations compared to their cisgender and heterosexual peers. This includes elevated rates of suicidal ideation and behavior, depression, anxiety, and higher rates of alcohol, tobacco, marijuana, and polysubstance use. Although more research is needed to assess when these disparities emerge across the life course, recent studies find LGBTQ+-related disparities in mental health as young as 10 years old and substance use as young as 12. These findings suggest that youth are aware of feeling different at a very young age and we need to consider how to support and affirm children who later understand themselves to be LGBTQ+. This could include simple alterations to classroom language and activities that acknowledge diverse family structures (e.g., having two moms or dads) and gender expression (e.g., avoid reinforcing specific play activities based on children’s assigned sex). Medical and mental health providers can also work with families to normalize children’s exploration of gender and gender expression. Acknowledging sexual orientation and gender diversity in a developmentally appropriate way can normalize these experiences and helps to eliminate shame and stigma.

Do we understand the reasons underlying these disparities?

Like other populations affected by health disparities, LGBTQ+ people—and transgender and gender diverse (TGD) people in particular—are susceptible to structural (e.g., laws and policies) and interpersonal (e.g., discrimination, harassment) stigma, which undermine their health and wellbeing. This includes barriers to accessing resources like housing and healthcare. For example, LGB youth who live in states with fewer protective policies (e.g., anti-bullying or anti-discrimination policies that name sexual orientation as a protected status) show higher rates of suicidal ideation and behavior. We also know that LGBTQ+ people who experience discrimination and victimization are more likely to report poorer mental health and greater substance use. Unfortunately, these experiences with stigma are typical for LGBTQ+ people and start early in the life course, often from family and peers.

What research is needed to better understand these disparities and how they impact LGBTQ+ people?

At this point, LGBTQ+-related health disparities and their link to stigma are well-established. What lags is the implementation of promising strategies to address—and, more importantly, prevent—these inequities across multiple sectors (e.g., education, healthcare, mental health services). For example, if LGBTQ+ -related disparities in mental health are present by age 10, we need to consider how schools can affirm and normalize sexual and gender diversity before middle school and high school. A requisite component to addressing LGBTQ+-related health inequities is to assure that medical and mental health professionals receive adequate training to work with LGBTQ+ clients, including LGBTQ+ youth and their families.

How does your own research further these goals?

A main focus of my research seeks to understand how experiences within schools, families, and communities shape the development and health of LGBTQ+ young people and inform strategies (e.g., policies, programs) that promote health. More recently, with the University of Maryland Prevention Research Center (UMD-PRC), I am working with researchers and clinicians to develop and evaluate a comprehensive training program for community mental health organizations and therapists to increase their cultural competence when serving LGBTQ+ clients. Compared to the general population, LGBTQ+ people are more likely to engage with mental healthcare services, but often report experiences with uninformed service providers who perpetuate stereotypes or cause harm in the therapeutic process. We are currently wrapping up a randomized controlled trial and are seeing some promising results regarding the effectiveness of our training program. We hope to be able to offer our training more broadly to help address the lack of required LGBTQ+ training in clinical graduate programs and circumvent the current services gap for LGBTQ+ populations. Generally, we need concerted research, advocacy, and training to address barriers to adequate mental and medical care for LGBTQ+ populations.

What changes do you think are needed, within healthcare or health policy, to begin to address these issues?

The current deluge of anti-transgender policies being proposed and enacted across the United States is a horrific example of how policy is being used to harm TGD young people. The science is clear^1,2^: access to gender-affirming care is associated with better mental health for TGD youth (and adults). Policymakers need to ensure that TGD youth have access to developmentally appropriate, gender-affirmative healthcare, including puberty suppression, gender-affirming hormones, and mental health support for TGD youth. Healthcare systems and providers can also advocate for insurance to cover developmentally appropriate, gender-affirmative healthcare. These efforts can be further supported by ensuring that medical providers receive adequate training in working with TGD clients so as not to perpetuate harm when providing services. Ultimately, LGBTQ+ people need policies to protect and ensure their rights to adequate and affirming healthcare. This will require substantive changes in provider training, service administration, and state-level policy. These efforts should be addressed in genuine partnership with LGBTQ+ community members and advocates.
